# Maternal vitamin B_12_ deficiency and perinatal outcomes in southern India

**DOI:** 10.1371/journal.pone.0248145

**Published:** 2021-04-06

**Authors:** Julia L. Finkelstein, Amy Fothergill, Jesse T. Krisher, Tinku Thomas, Anura V. Kurpad, Pratibha Dwarkanath

**Affiliations:** 1 Division of Nutritional Sciences, Cornell University, Ithaca, NY, United States of America; 2 St. John’s Research Institute, Bangalore, Karnataka, India; Holbaek Sygehus, DENMARK

## Abstract

**Background:**

Vitamin B_12_ deficiency during pregnancy has been associated with adverse maternal and infant health outcomes. Few prospective studies have investigated vitamin B_12_ status early in pregnancy, and its links to infant vitamin B_12_ status, particularly in India where the burden of vitamin B_12_ deficiency is estimated to be the highest globally. The objective of this study was to examine the associations of maternal vitamin B_12_ biomarkers with neonatal vitamin B_12_ status.

**Methods:**

Pregnant women (~12 weeks’ gestation) were enrolled in a perinatal cohort study in Bangalore, India. Total vitamin B_12_, methylmalonic acid (MMA), and homocysteine concentrations were evaluated in maternal samples at enrollment and in neonates at birth using cord blood. Linear and binomial regression models were used to evaluate the associations of maternal vitamin B_12_ biomarkers with neonatal vitamin B_12_ status and perinatal outcomes.

**Results:**

A total of 63.2% of women had vitamin B_12_ deficiency (<148 pmol/L), 87.2% had vitamin B_12_ insufficiency (<221 pmol/L), and 47.3% had impaired vitamin B_12_ status (vitamin B_12_<148 pmol/L and MMA>0.26μmol/L) at enrollment; 40.8% of neonates had vitamin B_12_ deficiency, 65.6% were insufficiency, and 38.1% had impaired vitamin B_12_ status at birth. Higher maternal vitamin B_12_ concentrations at enrollment were associated with increased neonatal vitamin B_12_ concentrations (β(SE): 0.40 (0.05); p<0.0001) and lower risk of neonatal vitamin B_12_ deficiency (Risk Ratio [RR]: 0.53; 95% CI: [0.43, 0.65]; p<0.0001). Maternal vitamin B_12_ deficiency (RR: 1.97 [1.43, 2.71]; p<0.001), insufficiency (RR: 2.18 [1.23, 3.85]; p = 0.007), and impaired vitamin B_12_ status (RR: 1.49 [1.13, 1.97]; p = 0.005) predicted a two-fold increase in the risk of neonatal vitamin B_12_ deficiency at birth.

**Conclusions:**

The prevalence of vitamin B_12_ deficiency was high early in pregnancy and predicted neonatal vitamin B_12_ status. Future research is needed to determine the role of vitamin B_12_ in the development of pregnancy and infant outcomes, and to inform screening and interventions to improve maternal and child health.

## Introduction

Vitamin B_12_ deficiency (vitamin B_12_ <148 pmol/L) is an important public health problem worldwide [1–3]. Although there is limited population-level data, vitamin B_12_ deficiency affects individuals across the life cycle, with the highest prevalence in the elderly, pregnant women, and young children [1, 2, 4–12]. The burden of vitamin B_12_ deficiency in India is estimated to be among the highest in the world [1, 13–23]. Inadequate vitamin B_12_ status during pregnancy has been associated with increased risk of adverse maternal and infant health outcomes [1–3, 24, 25], and linked to long-term impairments in child growth and development which may be irreversible [3, 24, 26–30].

Previous cross-sectional research has noted associations between maternal and infant vitamin B_12_ status at delivery in studies in Belgium, Canada, Norway, Germany, United Kingdom, Turkey, Serbia, and Brazil [3, 31–40]. Findings from prospective studies have reported significant associations of maternal vitamin B_12_ status during pregnancy with infant vitamin B_12_ status at birth (i.e., in cord blood or serum) [41–46] and at six weeks of age [23], in studies in the Netherlands, Norway, Turkey, Spain, India, and the United States. In contrast, findings regarding the associations of maternal vitamin B_12_ status with other child health outcomes or vitamin B_12_ status later in childhood have been heterogeneous. Inadequate maternal vitamin B_12_ status during gestation has also been associated with risk of pregnancy complications, such as spontaneous abortion, preterm delivery, intrauterine growth restriction, low birth weight, and neural tube defects [3, 7, 47–66]. However, most studies to date have been cross-sectional or case-control in design, constrained by limited sample sizes, and relied on a single biomarker of maternal total vitamin B_12_ concentrations, evaluated at mid-gestation or delivery.

Few prospective studies have been conducted to date to examine the impact of maternal vitamin B_12_ status during pregnancy on vitamin B_12_ status early in life, or the role of vitamin B_12_ in the development of adverse birth or infant outcomes [3]. There is limited prospective data, particularly early in gestation, and from settings with the highest burden of vitamin B_12_ deficiency and adverse pregnancy outcomes, such as India. Further research is needed to determine the burden of vitamin B_12_ deficiency in pregnant women and their infants in high-risk populations, and its implications for maternal and child health outcomes.

We conducted a prospective observational analysis to: 1) determine the prevalence of vitamin B_12_ deficiency in pregnant women and their infants; 2) examine the associations of maternal vitamin B_12_ biomarkers with neonatal vitamin B_12_ status; and 3) examine the associations of maternal vitamin B_12_ biomarkers with perinatal outcomes, in women participating in a cohort study in Bangalore, India.

## Methods

### Ethics statement

The research protocol and study procedures were approved by the Institutional Ethical Board of St. John’s Medical College (Reference number: IEC 42/2001). Written signed informed consent was obtained from all study participants at enrollment.

### Study population

**Fig 1:**
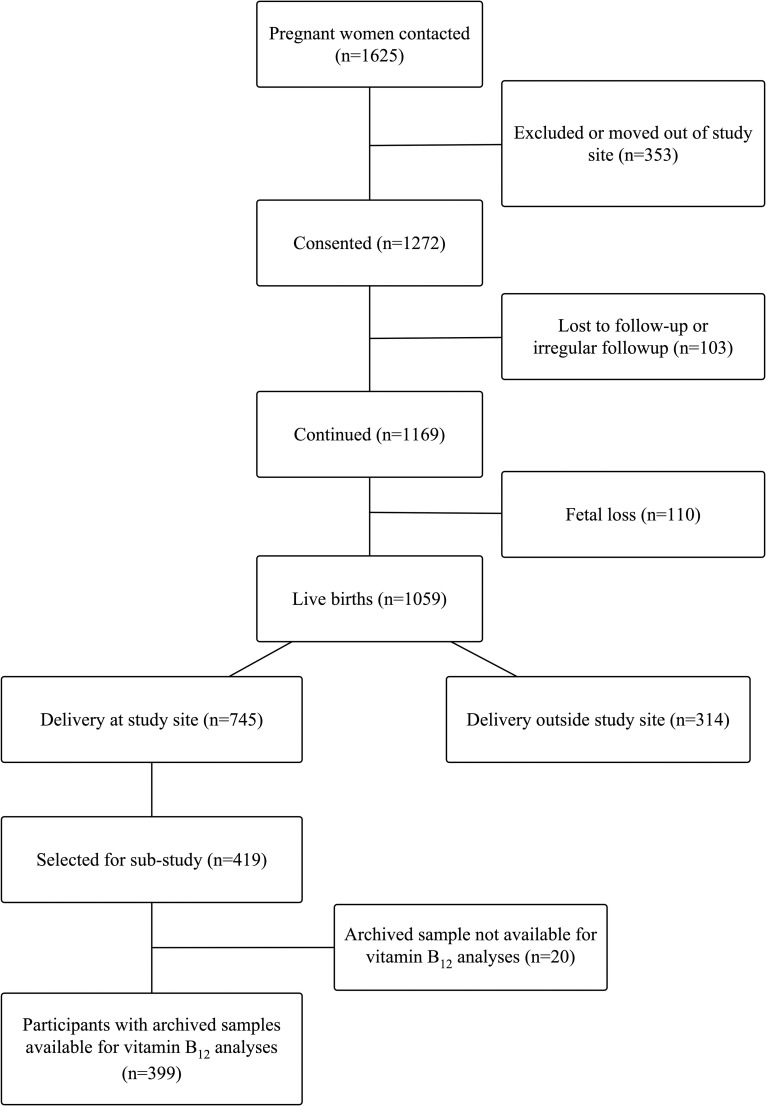
Participant flow chart.

This study was a prospective observational cohort study of pregnant women conducted at St John’s Medical College Hospital (SJMCH) in Bangalore, India. St. John’s Medical College is a 1350-bed teritary care hospital located in Bangalore, India. Established with a commitment to serve poor and vulnerable populations, its catchment includes a patient population from diverse social and economic backgrounds. The overall study design of the perinatal cohort and recruitment [67], inclusion criteria, and sampling for the biomarker sub-study [68] have been previously published. Pregnant women were eligible for this study if they were at least 17 years of age, <14 weeks of gestation at enrollment, healthy, and carrying a single fetus. Women who had multiple fetuses (e.g., twins, triplets), reported clinical diagnoses of a chronic condition (e.g., diabetes mellitus, hypertension, cardiovascular disease, thyroid disease), tested positive for HIV, hepatitis B, or syphilis infections, were taking medications, or planned to move outside of Bangalore prior to delivery were excluded from the study. Pregnant women recruited to the perinatal cohort study from 2008 to 2014 who had a venous blood sample collected at enrollment, delivered a live baby at SJCMH, and had cord blood collected at the time of delivery were eligible for this analysis (n = 419). A flow chart of study participants is presented in **Fig 1**.

All women received routine antenatal supplements in accordance with the National guidelines of India. As per standard of care, all women received folic acid at recruitment until the end of the first trimester, followed by iron-folic acid and calcium supplementation from the second trimester through delivery. Participants did not take vitamin B_12_ supplements or multivitamins containing vitamin B_12_, and no additional supplements or counseling were provided. All study procedures were reviewed and approved by the institutional ethical review board at SJCMH, and all participants provided written and signed consent at enrollment.

### Data collection

Validated structured questionnaires were administered by trained research staff to collect sociodemographic, clinical, and dietary data, and standardized forms were used to record anthropometric and biochemical data prospectively beginning at enrollment. Gestational age in weeks was calculated from the day of last menstrual period and confirmed through ultrasonographic measurements (GE Volusun 730 Expert, probe 4C-A, Via Del Rio, Yorba Linda, CA, USA) within two weeks of enrollment; ultrasonographic measurements were collected again before delivery.

At each antenatal visit, maternal weight was recorded to the nearest 100 grams using a digital balance (Soehnie, Reutilngen, Germany); maternal height was recorded to the nearest 0.1 centimeter using a stadiometer; mid-upper arm circumference (MUAC; cm) was recorded using a plastic tape; and skinfolds thickness (biceps, triceps, subscapular; mm) were recorded to the nearest 0.2 millimeters using Holtain skinfold calipers (Holtain Limited, Crosswell, Wales, UK). Maternal body mass index (BMI) was calculated as weight in kilograms divided by height in meters squared (kg/m^2^). Infant birth weight was measured on an electronic weighing scale (Salter Housewares 914 Electronic Baby and Toddler Scale, NY, USA) immediately after birth to the nearest 10 grams. Infant length (cm), circumferences (MUAC, head, chest; cm), and skinfolds (biceps, triceps, subscapular; mm) were measured within 72 hours of birth.

### Laboratory analyses

The laboratory procedures and analyses have been previously described [67, 68]. Briefly, maternal venous whole blood and cord blood samples were collected at enrollment and delivery, respectively, in ethylenediaminetetraacetic acid (EDTA)-coated anticoagulant tubes and plain vacutainers (Becton Dickenson, NJ, USA). Maternal venous whole blood and cord blood samples were processed and analyzed in batch, using identical protocols, and instrument specific calibrators were used for all biochemical estimations. Whole blood was treated with 1% ascorbic acid, and hemolysate was stored <–80°C. Plasma, serum, and red blood cells were separated, processed, and stored <–80°C until batch analysis. A total of 399 participants had blood samples at enrollment available for laboratory analyses.

Hemoglobin and complete blood count (CBC) were analyzed using an automated cyanmethemoglobin technique analyzer (ABX Pentra 60 C+, Horiba ABX Diagnostics, Montpellier, France). The measuring range was between 8 and 18 g/dL with a within run precision of <1.0%. Plasma vitamin B_12_ concentrations were measured *via* electrochemiluminescence (Elecsys 2010, Roche Diagnostics Mannheim, USA). Quality-control samples pertaining to low, middle, and high ranges of vitamin B_12_ were analyzed along with the samples. Intra- and inter-assay CVs were 0.5% and 2.4%, respectively. Plasma methylmalonic acid (MMA) and total homocysteine (tHcy) concentrations were assessed by gas chromatography-mass spectrometry (GCMS-SQ, 5975, Agilent Technologies, CA, USA) [69].

### Definitions of exposures and outcomes

Primary analyses were based on continuous vitamin B_12_ biomarkers (i.e., total vitamin B_12_, MMA) in pregnant women and neonates (i.e., cord blood). We also used conventional cut-offs from adult (non-pregnant) populations to describe categorical vitamin B_12_ variables. Vitamin B_12_ deficiency and insufficiency were defined as total vitamin B_12_ concentrations <148 and <221 pmol/L, respectively [7, 70]. Elevated methylmalonic acid concentrations were defined as MMA >0.26 and >0.37 μmol/L, to reflect depleted and deficient vitamin B_12_ status, respectively [7]. Impaired vitamin B_12_ status was defined as total vitamin B_12_ concentrations <148 pmol/L and MMA >0.26 μmol/L. Combined vitamin B_12_ (cB_12_), a composite indicator of vitamin B_12_ status, modified for three biomarkers (i.e., vitamin B_12_, MMA, tHcy), was calculated and defined using the methods developed by Fedosov et al. [71]. Elevated homocysteine concentrations were defined as tHcy >15.0 and >10.0 μmol/L [7]. Maternal anemia was defined as hemoglobin <11.0 g/dL [72].

Preterm delivery was defined as <37 weeks of completed gestation. Low birth weight was defined as birth weight <2,500 grams [73]. Small for gestational age (SGA) was defined as birth weight <10^th^ percentile for gestational age and sex, using the INTERGROWTH reference [73]. Infant ponderal index was defined as weight in grams divided by length in centimeters cubed (g/cm^3^). World Health Organization (WHO) standards were used to calculate length-for-age (LAZ), weight-for-age (WAZ), and weight-for-length (WLZ) z-scores. Stunting was defined as LAZ <–2, underweight as WAZ <–2, and wasting as WLZ <–2 [74–76]. Neonatal anemia was defined as hemoglobin <11.0 g/dL [72].

Birth outcomes (e.g., birth weight, low birth weight, gestational age at delivery, preterm delivery); neonatal vitamin B_12_ status (e.g., vitamin B_12_ concentrations, MMA concentrations, impaired vitamin B_12_ status) [77–79]; and neonatal anthropometric outcomes (e.g., ponderal index, LAZ, WAZ, WLZ, MUAC) were evaluated.

### Statistical analyses

Variables were defined using conventional cutoffs wherever available; medians were used to describe variables based on distributions in this population. Non-normally distributed variables were natural-logarithmically transformed to ensure normality before analysis. Non-transformed data are presented in **Tables 1** and **2** for interpretation purposes.

**Table 1:** Characteristics of the study population.

**Maternal Characteristics^1^**	**n**	**n (%) or Median (IQR)**
*Sociodemographic*		
Age, y	399	24.0 (21.0, 26.0)
Monthly household income, INR^2^	399	14,000 (10,000, 25,000)
<6,000 INR		45 (11.3)
Housing	399	
Kuccha (kachha)		12 (3.0)
Pucca		166 (41.6)
Thatched		203 (50.9)
Mixed / semi pucca		18 (4.5)
Education	399	
Up to high school		122 (30.6)
High school diploma (or pre-university or diploma)		134 (33.6)
University degree and above		143 (35.8)
Formal employment	399	79 (19.8)
Family type	399	
Nuclear		147 (36.8)
Extended		167 (41.9)
Joint		85 (21.3)
Gestational age at enrollment, weeks	399	12.0 (9.6, 13.3)
Parity	399	
Nulliparous		221 (55.4)
Primiparous or multiparous		178 (44.6)
Dietary Preference^3^		
Vegan	398	0 (0.0)
Vegetarian (i.e., milk and/or eggs)	398	71 (17.8)
Non-vegetarian (i.e., poultry, meat, and/or fish)	398	327 (82.2)
Prenatal Supplement Use^4^		
Trimester 1	378	266 (70.4)
Trimester 2	378	378 (100.0)
Trimester 3	378	377 (99.7)
*Anthropometric*		
Weight, kg	399	50.1 (44.9, 56.3)
Height, cm	399	156.2 (152.0, 160.0)
<150 cm		66 (16.5)
Body mass index^5^, kg/m^2^	399	20.6 (18.6, 23.4)
<18.5 kg/m^2^		94 (23.6)
18.5 to <25.0 kg/m^2^		248 (62.2)
25.0 to <30.0 kg/m^2^		52 (13.0)
≥30.0 kg/m^2^		5 (1.3)
Body mass index (alternate)^6^, kg/m^2^	399	20.6 (18.6, 23.4)
<18.5 kg/m^2^		94 (23.6)
18.5 to <23.0 kg/m^2^		193 (48.4)
23.0 to <27.5 kg/m^2^		96 (24.1)
≥27.5 kg/m^2^		16 (4.0)
Mid-upper arm circumference, cm	399	23.5 (21.7, 25.8)
*Biochemical (Enrollment)*		
Hemoglobin, g/dL	282	11.9 (11.1, 12.7)
<11.0 g/dL		63 (22.3)
Packed cell volume	399	34.2 (31.9, 36.4)
**Birth Outcomes**^**1**^	**n**	**n (%) or Median (IQR)**
Birthweight, g	399	2920 (2660, 3180)
<2,500 g		54 (13.5)
Gestational age at birth, weeks	399	39.0 (38.0, 39.5)
<37 weeks		24 (6.0)
Small for gestational age^7^	399	86 (21.6)
**Neonatal Outcomes**^**1**^	**n**	**n (%) or Median (IQR)**
*Biochemical*		
Hemoglobin, g/dL	252	14.3 (12.4, 15.6)
<11.0 g/dL		38 (15.1)
*Anthropometric*		
Length, cm	389	49.7 (48.7, 50.7)
Length-for-age z-score (LAZ)	389	–0.01 (–0.56, 0.53)
Stunting (LAZ <–2)		15 (3.9)
Weight-for-age z-score (WAZ)	399	–0.74 (–1.36, –0.15)
Underweight (WAZ <–2)		39 (9.8)
Weight-for-length z-score (WLZ)	384	–1.44 (–2.30, –0.42)
Wasting (WLZ <–2)		131 (34.1)
Ponderal index^8^, kg/m^3^	389	0.024 (0.022, 0.026)
Head circumference, cm	391	33.5 (32.5, 34.2)
Chest circumference, cm	390	31.5 (30.4, 32.7)
Mid-upper arm circumference, cm	391	9.7 (9.1, 10.2)
Biceps skinfold, mm	390	3.0 (2.6, 3.4)
Triceps skinfold, mm	390	3.9 (3.2, 4.4)
Subscapular skinfold, mm	390	4.2 (3.6, 4.9)

^1^Sample size (n) changes by row due to data availability, reported values are median (IQR) and n (%)

^2^100 INR was equivalent to approximately US$2 at the time the study was conducted

^3^Vegan: Does not consume milk, eggs, poultry, meat, or fish; vegetarian: Consumes milk and/or eggs, but not poultry, meat, or fish; non-vegetarian: Consumes poultry, meat, and/or fish

^4^All participants were prescribed routine antenatal supplements as part of National guidelines of India; prenatal supplements did not include vitamin B_12_.

^5^BMI categories as defined by the WHO [80]

^6^BMI categories for Asian populations [81]

^7^Small for gestational age was defined as birth weight <10^th^ percentile for gestational age and sex, using INTERGROWTH reference [73]

^8^Neonatal ponderal index was calculated as the ratio of weight to length (g/cm^3^ × 100); *Abbreviations*: INR, Indian rupees.

**Table 2:** Maternal vitamin B_12_ status at enrollment and neonatal vitamin B_12_ status at delivery.

	n	Maternal^1^	n	Neonatal^1^	P-value^4^
Plasma vitamin B_12_, pmol/L	399	127.0 (89.6, 172.3)	355	172.4 (109.7, 265.1)	<0.0001*
<148 pmol/L		252 (63.2)		145 (40.8)	<0.0001*
<221 pmol/L		348 (87.2)		233 (65.6)	<0.0001*
Plasma MMA, μmol/L	376	0.38 (0.24, 0.59)	374	0.63 (0.47, 0.86)	<0.0001*
>0.26 μmol/L		268 (71.3)		358 (95.7)	<0.0001*
>0.37 μmol/L		194 (51.6)		327 (87.4)	<0.0001*
Plasma tHcy, μmol/L	377	15.7 (12.4, 20.7)	374	17.6 (13.5, 24.8)	<0.0001*
>15.0 μmol/L		206 (54.6)		248 (66.3)	0.001*
>10.0 μmol/L		334 (88.6)		337 (90.1)	0.50
Impaired vitamin B_12_ status^2^	376	178 (47.3)	331	126 (38.1)	0.01
cB12^3^	376	–1.1 (–1.6, –0.7)	331	–1.4 (–1.8, –1.0)	<0.0001*
≥1.5		0 (0.0)		0 (0.0)	<0.0001*
–0.5 to <1.5		70 (18.6)		32 (9.7)	
–1.5 to <–0.5		195 (51.9)		147 (44.4)	
–2.5 to <–1.5		107 (28.5)		136 (41.1)	
<–2.5		4 (1.1)		16 (4.8)	

^1^Sample size (n) changes by row due to data availability, reported values are median (IQR) and n (%)

^2^Impaired vitamin B_12_ status: Plasma vitamin B_12_ <148 pmol/L and MMA >0.26 μmol/L

^3^cB12, a combined indicator of vitamin B_12_ status modified for three biomarkers (i.e., vitamin B_12_, MMA, tHcy), was calculated and defined using the methods developed by Fedosov [71]

^4^P-values are from Kruskal Wallis test for continuous and Chi-Sq test for categorical comparisons

*After adjusting for multiple hypothesis testing, associations were considered significant if p<0.004

*Abbreviations*: MMA, methylmalonic acid; tHcy, total homocysteine.

Primary analyses were based on continuous vitamin B_12_ biomarkers (i.e., total vitamin B_12_, MMA) in pregnant women and neonates (i.e., cord blood). We also used conventional cut-offs from adult (non-pregnant) populations to define categorical vitamin B_12_ variables. Linear and binomial regression models were used to evaluate the associations of maternal vitamin B_12_ biomarkers at enrollment with neonatal vitamin B_12_ status and perinatal outcomes, for continuous and categorical outcomes, respectively. Binomial regression models were used to obtain risk ratio estimates for dichotomous variables, and Poisson regression models were used when binomial regression models did not converge [77, 79]. All models were adjusted for gestational age at sample collection, in order to account for timing of sample collection. In order to adjust for multiple hypothesis testing, significance was evaluated after applying the Bonferroni correction. All p-values presented are the original (unadjusted) p-values for interpretation purposes, and the threshold used to determine statistical significance was α/n, where α is the level of significance (α = 0.05) and n is the number of comparison tests conducted. If the results remained significant after applying the Bonferroni correction, this is reported in the text.

The Rothman and Greenland approach was used to evaluate and adjust for confounding, in which all known or suspected risk factors for the outcome which led to a >10% change-in-estimate were included in the model [59]. Final linear models were assessed for normality, using the Kolmogorov-Smirnoff test; collinearity, using variance inflation factors; and homoscedasticity, using plots of residuals versus predicted values. Final binomial models were examined for goodness of fit, using Hosmer-Lemeshow tests. Statistical analyses were conducted using SAS software, version 9.4 (SAS Institute, Inc., Cary, NC, USA).

## Results

### Participant characteristics

A flowchart of participants in this study is presented in **Fig 1**. The design of the perinatal cohort study [67] and inclusion criteria and sampling for the biomarker sub-study [68] have been previously published. Briefly, a total of 1,625 pregnant women were initially contacted regarding the perinatal cohort study, of which 1,272 provided informed consent. There were a total of 1,059 live births (n = 103 lost to follow-up; n = 110 fetal loss), of which 745 participants delivered at the study site, SJCMH. A total of 419 participants were selected for the biomarker sub-study [68]; of these, vitamin B_12_ status was analyzed in 399 maternal enrollment samples (n = 20 insufficient sample/sample volume). Birth outcome data was available for 399 neonates, and 374 cord blood samples were available for vitamin B_12_ analyses.

The characteristics of participants in this study are presented in **Table 1**. Women selected for the biomarker sub-study (n = 419) were similar compared to women in the overall cohort (n = 1,272 consented) with respect to baseline characteristics, including age, gestational age at enrollment, socioeconomic status (e.g., education), parity, and nutritional indicators (e.g., weight, hemoglobin). At enrollment, women had a median age of 24.0 (IQR: 21.0, 26.0) years, and median gestational age of 12.0 (IQR: 9.6, 13.3) weeks; 55.4% were nulliparous and 35.8% had received a university degree. A total of 82.2% were non-vegetarian (i.e., consumed poultry, meat, and/or fish), 17.8% of women were vegetarian (i.e., consumed milk and/or eggs), and 0% were vegan (i.e., no animal source foods); 70.4% of women reported taking prenatal supplements at enrollment. Participants did not take vitamin B_12_ supplements or multivitamins containing vitamin B_12_. At delivery, 13.5% of neonates were low birthweight (<2,500 g), 6.0% were preterm (<37 weeks’ gestation), and 21.6% were small for gestational age (**Table 1**).

### Maternal vitamin B_12_ status

Vitamin B_12_ status in pregnant women at enrollment is presented in **Table 2**. At enrollment (median [IQR]: 12.0 [9.6, 13.3] weeks), 63.2% of women had vitamin B_12_ deficiency (vitamin B_12_ <148 pmol/L), 87.2% had vitamin B_12_ insufficiency (vitamin B_12_ <221 pmol/L), 71.3% had elevated methylmalonic acid (MMA >0.26 μmol/L) levels (MMA >0.37 μmol/L: 51.6%), and 47.3% had impaired vitamin B_12_ status (vitamin B_12_ <148 pmol/L and MMA >0.26 μmol/L). Associations between maternal biomarkers of vitamin B_12_ status (i.e., MMA, tHcy) and maternal vitamin B_*12*_ deficiency are presented in **S1 Table.** Maternal MMA levels (RR: 1.12; 95% CI: [0.43, 0.65]; p = 0.04) and elevated MMA (>0.26 μmol/L; RR: 1.26 [1.03, 1.53]; p = 0.02) were associated with maternal vitamin B_12_ deficiency.

### Neonatal vitamin B_12_ status

Vitamin B_12_ status in neonates at birth is presented in **Table 2**. A total of 40.8% of neonates had vitamin B_12_ deficiency (vitamin B_12_ <148 pmol/L), 65.6% had vitamin B_12_ insufficiency (vitamin B_12_ <221 pmol/L), 95.7% had elevated MMA (MMA >0.26 μmol/L) concentrations (MMA >0.37 μmol/L: 87.4%), and 38.1% had impaired vitamin B_12_ status (vitamin B_12_ <148 pmol/L and MMA >0.26 μmol/L) at birth. Associations between neonatal biomarkers of vitamin B_12_ status (i.e., MMA, tHcy) and neonatal vitamin B_12_ deficiency are presented in S1 Table. Neonatal MMA levels (RR: 1.59 [1.25, 2.02]; p = 0.0001 and tHcy concentrations (RR: 1.37 [1.06, 1.76]; p = 0.01) were associated with neonatal vitamin B_12_ deficiency.

### Maternal vitamin B_12_ status at enrollment and neonatal vitamin B_12_ status at birth

Vitamin B_12_ status in pregnant women at enrollment and in neonates at birth is shown in **Table 2**. Neonatal vitamin B_12_ concentrations were significantly higher compared to maternal vitamin B_12_ concentrations (172.4 [109.7, 265.1] vs. 127.0 [IQR: 89.6, 172.3]; p<0.0001). However, neonatal MMA (0.63 [IQR: 0.47, 0.86] vs. 0.38 [IQR: 0.24, 0.59]; p<0.0001) and homocysteine (17.6 [IQR: 13.5, 24.8] vs. 15.7 [IQR: 12.4, 20.7]; p<0.0001) levels were significantly higher in neonates. The prevalence of neonatal vitamin B_12_ deficiency (40.8% vs. 63.2%; p<0.0001), vitamin B_12_ insufficiency (65.6% vs. 87.2%; p<0.0001), and impaired vitamin B_12_ status (vitamin B_12_ <148 pmol/L and MMA >0.26 μmol/L; (38.1% vs. 47.3%; p = 0.01) were lower at delivery compared to pregnant women at enrollment.

The associations of maternal vitamin B_12_ biomarkers at enrollment with neonatal vitamin B_12_ concentrations at delivery are presented in **Table 3**. Higher maternal vitamin B_12_ concentrations were associated with increased neonatal vitamin B_12_ concentrations at delivery (β [SE]: 0.40 [0.05]; p<0.0001), in multivariate models adjusting for gestational age at enrollment, maternal age, parity, educational level, and BMI at enrollment. Maternal vitamin B_12_ deficiency (vitamin B_12_ <148 pmol/L; β [SE]: -0.30 [0.07]; p<0.0001) and vitamin B_12_ insufficiency (vitamin B_12_ <221 pmol/L; β [SE]: -0.54 [0.10]; p<0.0001) also predicted significantly lower neonatal vitamin B_12_ concentrations at delivery. Impaired maternal vitamin B_12_ status (vitamin B_12_ <148 pmol/L and MMA >0.26 μmol/L) at enrollment was associated with significantly lower neonatal vitamin B_12_ concentrations at delivery (β [SE]: –0.21 [0.07]; p<0.002) in multivariate analyses. Higher maternal cB_12_ was associated with increased neonatal vitamin B_12_ concentrations (β [SE]: 0.20 [0.05]; p<0.0001). Findings from these analyses remained statistically significant after correcting for multiple hypothesis testing (p<0.006). However, individually, maternal MMA, or homocysteine were not significantly associated with neonatal vitamin B_12_ concentrations at delivery.

**Table 3:** Associations between maternal vitamin B_12_ status at enrollment and neonatal vitamin B_12_ concentrations.

		Univariate^3^		Multivariate^4^	
Maternal Variables^1^^,^^2^	n	β (SE)	P-value	β (SE)	P-value^7^
Plasma vitamin B_12_, pmol/L	355	0.39 (0.05)	**<0.0001**	0.40 (0.05)	<0.0001^*^
<148 pmol/L		–0.30 (0.07)	**<0.0001**	–0.30 (0.07)	<0.0001^*^
<221 pmol/L		–0.53 (0.09)	**<0.0001**	–0.54 (0.10)	<0.0001^*^
Plasma MMA, μmol/L	332	–0.03 (0.05)	0.55	–0.03 (0.05)	0.59
>0.26 μmol/L		–0.01 (0.08)	0.85	–0.01 (0.07)	0.87
Impaired vitamin B_12_ status^5^	332	–0.22 (0.07)	**0.001**	–0.21 (0.07)	0.002^*^
Plasma tHcy, μmol/L	333	0.02 (0.09)	0.84	0.02 (0.09)	0.85
>15.0, μmol/L		–0.01 (0.07)	0.92	–0.01 (0.07)	0.87
cB_12_^6^	332	0.20 (0.05)	**<0.0001**	0.20 (0.05)	<0.0001^*^

^1^Sample size (n) changes by row due to data availability

^2^Statistical analyses: Linear regression models were used to examine associations between maternal biomarkers and neonatal vitamin B_12_ concentrations. Maternal biomarkers were natural logarithmically transformed to achieve normality prior to analysis

^3^Adjusted for gestational age at enrollment

^4^Adjusted for gestational age at enrollment, parity, maternal age in years, BMI, and educational level

^5^ Impaired vitamin B_12_ status: Vitamin B_12_ <148 pmol/L and MMA >0.26 μmol/L

^6^cB_12_, a combined indicator of vitamin B_12_ status modified for three biomarkers (i.e., vitamin B_12_, MMA, tHcy), was calculated using the method developed by Fedosov

^7*^Remained statistically significant after correcting for multiple hypothesis testing (p<0.006). *Abbreviations*: MMA, methylmalonic acid; tHcy, total homocysteine.

Maternal vitamin B_12_ biomarkers at enrollment and their associations with risk of neonatal vitamin B_12_ deficiency (vitamin B_12_ <148 pmol/L) are presented in **Table 4**. Higher maternal vitamin B_12_ concentrations predicted lower risk of neonatal vitamin B_12_ deficiency at birth (RR: 0.53 95% CI: [0.43, 0.65]; p<0.0001), in multivariate models adjusting for gestational age at enrollment, maternal age, parity, educational level, and BMI at enrollment. Impaired maternal vitamin B_12_ status (RR: 1.49 95% CI: [1.13, 1.97]; p = 0.005) and maternal vitamin B_12_ deficiency (RR: 1.97 95% CI: [1.43, 2.71]; p<0.0001) predicted a 1.49 to 1.97-fold higher risk of neonatal vitamin B_12_ deficiency at birth. Findings from these analyses remained statistically significant after correcting for multiple hypothesis testing (p<0.006). However, maternal MMA or homocysteine concentrations were not significantly associated with risk of neonatal vitamin B_12_ deficiency.

**Table 4:** Associations between maternal vitamin B_12_ status at enrollment and neonatal vitamin B_12_ deficiency (<148 pmol/L).

		Univariate^3^		Multivariate^4^	
Maternal Variables^1^^,^^2^	n	RR (95% CI)	P-value	RR (95% CI)	P-value^7^
Plasma vitamin B_12,_ pmol/L	355	0.54 (0.44, 0.66)	**<0.0001**	0.53 (0.43, 0.65)	<0.0001^*^
<148 pmol/L		2.00 (1.45, 2.75)	**<0.0001**	1.97 (1.43, 2.71)	<0.0001^*^
<221 pmol/L		2.16 (1.23, 3.81)	0.008	2.18 (1.23, 3.85)	0.007
Plasma MMA, μmol/L	332	1.02 (0.84, 1.25)	0.81	1.01 (0.83, 1.24)	0.92
>0.26 μmol/L		1.02 (0.76, 1.37)	0.90	0.99 (0.73, 1.33)	0.93
Impaired vitamin B_12_ status^5^	332	1.54 (1.18, 2.02)	**0.002**	1.49 (1.13, 1.97)	0.005^*^
Plasma tHcy, μmol/L	333	0.99 (0.70, 1.39)	0.95	0.98 (0.69, 1.38)	0.90
>15.0, μmol/L		0.99 (0.76, 1.30)	0.96	0.99 (0.76, 1.29)	0.95
cB_12_^6^	332	0.72 (0.59, 0.88)	**0.001**	0.74 (0.60, 0.90)	0.003^*^

^1^Sample size (n) changes by row due to data availability

^2^Statistical analyses: Binomial regression models were used to examine associations between maternal biomarkers and neonatal vitamin B_12_ deficiency. Poisson regression models were used when binomial regression models did not converge. Maternal biomarkers were natural logarithmically transformed to achieve normality prior to analysis

^3^Adjusted for gestational age at enrollment

^4^Adjusted for gestational age at enrollment, parity, and maternal age in years, BMI, and educational level

^5^ Impaired vitamin B_12_ status: Vitamin B_12_ <148 pmol/L and MMA >0.26 μmol/L

^6^cB_12_, a combined indicator of vitamin B_12_ status modified for three biomarkers (vitamin B_12_, MMA, tHcy), was calculated using method developed by Fedosov [71]

^7*^Remained statistically significant after correcting for multiple hypothesis testing (p<0.006). *Abbreviations*: MMA, methylmalonic acid; tHcy, total homocysteine.

The associations of maternal vitamin B_12_ biomarkers at enrollment with neonatal methylmalonic acid concentrations at delivery are shown in **Table 5.** Maternal vitamin B_12_ deficiency (β [SE]: 0.16 [0.05]; p = 0.002) at enrollment was associated with increased neonatal MMA concentrations at birth, in multivariate models adjusting for gestational age at enrollment, maternal age, parity, educational level, and BMI at enrollment. However, maternal vitamin B_12_ concentrations or impaired vitamin B_12_ status were not significantly associated with neonatal MMA concentrations at birth, after correcting for multiple hypothesis testing (p<0.006).

**Table 5:** Associations between maternal vitamin B_12_ status at enrollment and neonatal methylmalonic acid concentrations.

		Univariate^3^		Multivariate^4^	
Maternal Variables^1^^,^^2^	*n*	β (SE)	P-value	β (SE)	P-value^7^
Plasma vitamin B_12_, pmol/L	374	–0.10 (0.04)	0.02	–0.10 (0.04)	0.03
<148 pmol/L		0.16 (0.05)	**0.002**	0.16 (0.05)	0.002^*^
<221 pmol/L		0.11 (0.07)	0.15	0.11 (0.07)	0.14
Plasma MMA, μmol/L	367	0.01 (0.04)	0.77	0.01 (0.04)	0.75
>0.26 μmol/L		0.03 (0.06)	0.63	0.03 (0.06)	0.54
Impaired vitamin B_12_ status^5^	367	0.11 (0.05)	0.02	0.12 (0.05)	0.02
Plasma tHcy, μmol/L	368	–0.05 (0.07)	0.43	–0.06 (0.07)	0.35
>15.0, μmol/L		–0.01 (0.05)	0.80	–0.01 (0.05)	0.79
cB_12_^6^	367	–0.04 (0.04)	0.29	–0.04 (0.04)	0.33

^1^Sample size (n) changes by row due to data availability

^2^Statistical analyses: Linear regression models were used to examine associations between maternal biomarkers and neonatal MMA concentrations. Maternal biomarkers were natural logarithmically transformed to achieve normality prior to analysis

^3^Adjusted for gestational age at enrollment

^4^Adjusted for gestational age at enrollment, parity, and maternal age in years, BMI, and educational level

^5^Impaired vitamin B_12_ status: Vitamin B_12_ <148 pmol/L and MMA >0.26 μmol/L

^6^cB_12_, a combined indicator of vitamin B_12_ status modified for three biomarkers (vitamin B_12_, MMA, tHcy), was calculated using the method developed by Fedosov [71]

^7*^Remained statistically significant after correcting for multiple hypothesis testing (p<0.006). *Abbreviations*: MMA, methylmalonic acid; tHcy, total homocysteine.

The associations of maternal vitamin B_12_ biomarkers at enrollment with impaired neonatal vitamin B_12_ status at birth are presented in **Table 6**. Higher maternal vitamin B_12_ concentrations were associated with lower risk of impaired neonatal vitamin B_12_ status (RR: 0.52 95% CI: [0.42, 0.65]; p<0.0001), in multivariate analyses adjusting for gestational age at enrollment, maternal age, parity, educational level, and BMI at enrollment. Similarly, maternal vitamin B_12_ deficiency (RR: 2.08 95% CI: [1.46, 2.97]; p<0.0001) and impaired maternal vitamin B_12_ status (RR: 1.54 95% CI: [1.15, 2.06]; p = 0.004) predicted increased risk of impaired neonatal vitamin B_12_ status. Higher maternal cB_12_ (RR: 0.73 95% CI: [0.60, 0.90]; p = 0.004) was associated with lower risk of impaired neonatal vitamin B_12_ status at birth. Findings from these analyses remained statistically significant after correcting for multiple hypothesis testing (p<0.006).

**Table 6:** Associations between maternal vitamin B_12_ status at enrollment and impaired neonatal vitamin B_12_ status.

		Univariate^3^		Multivariate^4^	
Maternal Variables^1^^,^^2^	n	RR (95% CI)	P-value	RR (95% CI)	P-value^7^
Plasma vitamin B_12,_ pmol/L	331	0.53 (0.43, 0.66)	**<0.0001**	0.52 (0.42, 0.65)	<0.0001^*^
<148 pmol/L		2.11 (1.49, 3.00)	**<0.0001**	2.08 (1.46, 2.97)	<0.0001^*^
<221 pmol/L		2.15 (1.18, 3.93)	0.01	2.18 (1.19, 4.00)	0.01
Plasma MMA, μmol/L	324	1.03 (0.83, 1.27)	0.79	1.01 (0.82, 1.25)	0.91
>0.26 μmol/L		1.03 (0.76, 1.41)	0.84	1.01 (0.74, 1.38)	0.94
Impaired vitamin B_12_ status^5^	324	1.57 (1.18, 2.08)	**0.002**	1.54 (1.15, 2.06)	0.004^*^
Plasma tHcy, μmol/L	325	1.05 (0.74, 1.48)	0.80	1.03 (0.72, 1.47)	0.88
>15.0, μmol/L		1.06 (0.80, 1.41)	0.68	1.05 (0.79, 1.39)	0.74
cB_12_^6^	324	0.72 (0.59, 0.89)	**0.002**	0.73 (0.60, 0.90)	0.004^*^

^1^Sample size (n) changes by row due to data availability

^2^Statistical analyses: Binomial regression models were used to examine associations between maternal biomarkers and impaired neonatal vitamin B_12_ status. Poisson regression models were used when binomial regression models did not converge. Maternal biomarkers were natural logarithmically transformed to achieve normality prior to analysis

^3^Adjusted for gestational age at enrollment

^4^Adjusted for gestational age at enrollment, parity, and maternal age in years, BMI, and educational level

^5^Impaired vitamin B_12_ status: Vitamin B_12_ <148 pmol/L and MMA >0.26 μmol/L

^6^cB_12_, a combined indicator of vitamin B_12_ status modified for three biomarkers (i.e., vitamin B_12_, MMA, tHcy), was calculated using the method developed by Fedosov [71]

^7^*Remained statistically significant after correcting for multiple hypothesis testing (p<0.006). *Abbreviations*: MMA, methylmalonic acid; tHcy, total homocysteine.

Maternal vitamin B_12_ status at baseline and its associations with neonatal homocysteine levels at birth are summarized in **Table 7**. Maternal vitamin B_12_ insufficiency (vitamin B_12_ <221 pmol/L) at enrollment was associated with higher neonatal homocysteine concentrations (β [SE]: 0.22 [0.07]; p = 0.003) at birth, in multivariate analyses adjusting for gestational age at enrollment, parity, maternal age, BMI, and educational level at enrollment.

**Table 7:** Associations between maternal vitamin B_12_ status at enrollment and neonatal homocysteine concentrations.

		Univariate^3^		Multivariate^4^	
Maternal Variables^1^^,^^2^	n	β (SE)	P-value	β (SE)	P-value^7^
Plasma vitamin B_12,_ pmol/L	374	-0.08 (0.04)	0.08	-0.07 (0.04)	0.10
<148 pmol/L		0.06 (0.05)	0.25	0.06 (0.05)	0.23
<221 pmol/L		0.22 (0.07)	**0.003**	0.22 (0.07)	0.003^*^
Plasma MMA, μmol/L	367	0.08 (0.04)	0.04	0.08 (0.04)	0.03
>0.26 μmol/L		0.11 (0.06)	0.056	0.11 (0.06)	0.04
Impaired vitamin B_12_ status^5^	367	0.06 (0.05)	0.25	0.06 (0.05)	0.21
Plasma tHcy, μmol/L	368	0.06 (0.07)	0.39	0.05 (0.07)	0.46
>15.0, μmol/L		0.01 (0.05)	0.89	0.01 (0.05)	0.91
cB_12_^6^	367	-0.10 (0.04)	0.01	-0.09 (0.04)	0.01

^1^Sample size (n) changes by row due to data availability

^2^Statistical analyses: Linear regression models were used to examine associations between maternal biomarkers and neonatal homocysteine concentrations. Maternal biomarkers were natural logarithmically transformed to achieve normality prior to analysis

^3^Adjusted for gestational age at enrollment

^4^Adjusted for gestational age at enrollment, parity, and maternal age in years, BMI, and educational level

^5^Impaired vitamin B_12_ status: Plasma vitamin B_12_ <148 pmol/L and MMA >0.26 μmol/L

^6^cB_12_, a combined indicator of vitamin B_12_ status modified for 3 biomarkers (vitamin B_12_, MMA, tHcy), was calculated using the method developed by Fedosov [71]

^7*^Remained statistically significant after correcting for multiple hypothesis testing (p<0.006). *Abbreviations*: MMA, methylmalonic acid; tHcy, total homocysteine.

### Maternal vitamin B_12_ status at enrollment and perinatal outcomes

The associations between maternal vitamin B_12_ biomarkers at enrollment with perinatal outcomes are presented in supplemental tables (**S2–S4 Tables)**. After adjusting for gestational age at enrollment, parity, maternal age, BMI, and educational level at enrollment, maternal vitamin B_12_ concentrations were not significantly associated with perinatal outcomes (S2 Table). Similarly, maternal MMA concentrations at enrollment were not associated with perinatal outcomes in multivariate analyses (S3 Table). After adjusting for gestational age at enrollment, parity, maternal age, BMI, and highest level of education attained, impaired maternal vitamin B_12_ status (vitamin B_12_ <148 pmol/L and MMA >0.26 μmol/L) at enrollment was not associated with any perinatal outcomes (S4 Table). Other maternal vitamin B_12_ biomarkers were not significantly associated with perinatal outcomes, after correcting for multiple hypothesis testing (p<0.002).

## Discussion

In this prospective analysis among pregnant women participating in a cohort study, maternal vitamin B_12_ deficiency was common early in pregnancy and predicted neonatal vitamin B_12_ status at birth. Maternal vitamin B_12_ status at enrollment–including vitamin B_12_ deficiency (<148 pmol/L), insufficiency (<221 pmol/L), and impaired vitamin B_12_ status (vitamin B_12_ <148 pmol/L and MMA >0.26 μmol/L)—predicted risk of neonatal vitamin B_12_ deficiency. Higher maternal vitamin B_12_ concentrations at enrollment were associated with increased neonatal vitamin B_12_ concentrations. Maternal vitamin B_12_ status at enrollment was not associated with risk of other perinatal outcomes.

This is among the largest prospective studies conducted to date to examine the burden of vitamin B_12_ deficiency in pregnancy and its associations with neonatal vitamin B_12_ status. Vitamin B_12_ deficiency was common early in pregnancy: 63.2% of women had vitamin B_12_ concentrations <148 pmol/L, 87.2% had vitamin B_12_ levels <221 pmol/L, and 47.3% had impaired vitamin B_12_ status (i.e., vitamin B_12_ <148 pmol/L and MMA >0.26 μmol/L) at enrollment. Findings are consistent with previous studies conducted among pregnant women in India (vitamin B_12_ <148 pmol/L or <162 pmol/L: 51–73%; ≤18 weeks’ gestation) [20, 23, 82, 83], and higher than studies in Bangladesh (vitamin B_12_ <150 pmol/L: 35.4%; <13 weeks’ gestation), Spain (vitamin B_12_ <150 pmol/L: 0%; ≤221 pmol/L: 6.3%; <12 weeks’ gestation), and Canada (vitamin B_12_ <148 pmol/L: 17%; 12–16 weeks’ gestation) [12, 39, 43, 84, 85].

The prevalence of vitamin B_12_ deficiency and insufficiency was also high in neonates at birth in this study. A total of 41% of neonates had vitamin B_12_ deficiency, 66% had vitamin B_12_ insufficiency, and 38% had impaired vitamin B_12_ status at birth. Findings are consistent with previous studies conducted among young infants in India (vitamin B_12_ <150 pmol/L: 44%, 6 weeks; <150 pmol/L: 62%, 1–3 months of age) [23, 86] and higher than in studies conducted in the UK (vitamin B_12_ <140.9 pmol/L: 29%; cord blood at delivery) and the United States (<148 pmol/L: 0%; cord blood at delivery) [45]. In the current study, neonatal vitamin B_12_ concentrations were 1.3-fold higher than maternal vitamin B_12_ concentrations early in pregnancy. Findings are consistent with previous studies that reported higher vitamin B_12_ concentrations in infants compared to mothers, ranging from 13 to 43% at mid-gestation [23, 42, 44, 46, 87, 88] or delivery [31–40, 43, 45, 89] to two-fold higher than maternal vitamin B_12_ concentrations at delivery [35, 36, 43, 45].

In the current study, maternal vitamin B_12_ status early in pregnancy–including vitamin B_12_ concentrations, vitamin B_12_ deficiency and insufficiency, and impaired vitamin B_12_ status–predicted neonatal vitamin B_12_ status at birth. For example, neonates born to women who had vitamin B_12_ deficiency at enrollment had a 2-fold greater risk of vitamin B_12_ deficiency at birth. Findings are consistent with studies of the associations of maternal vitamin B_12_ concentrations during gestation [23, 42, 44, 87, 88] and at delivery [31–38, 40, 43, 45, 89] with neonatal vitamin B_12_ levels at birth and in the first six weeks of life.

Maternal vitamin B_12_ deficiency at enrollment also predicted neonatal MMA concentrations and elevated neonatal MMA concentrations at birth. Few studies to date have evaluated MMA concentrations or other functional biomarkers of vitamin B_12_ status in young infants, and findings have been divergent. For example, in a study in Ireland, maternal vitamin B_12_ levels at 8 weeks of gestation were associated with maternal MMA concentrations during labor, but not with MMA levels in cord blood [43]. However, this study was constrained by limited range of maternal vitamin B_12_ status (i.e., no participants had vitamin B_12_ deficiency or elevated MMA concentrations during pregnancy; and ~26% reported taking cobalamin containing supplements during pregnancy) and smaller sample size (n = 92). In a study in Norway among 169 mother-infant pairs postpartum, maternal vitamin B_12_ levels were significantly correlated with infant MMA levels (r = -0.38, p<0.001) [90]; however, this study did not report MMA levels during pregnancy to which our findings can be directly compared.

In the current study, maternal vitamin B_12_ status during pregnancy was not significantly associated with risk of adverse perinatal outcomes. Findings are in contrast to previous research which identified vitamin B_12_ as a risk factor for adverse birth outcomes [3]: inadequate maternal vitamin B_12_ status in pregnancy has been associated with increased risk of spontaneous abortion or early miscarriage [54, 55, 91, 92], low birth weight (LBW, <2,500 g) [57, 83, 93, 94], intrauterine growth restriction (IUGR) [93], small for gestational age (SGA) [94], and neural tube defects [48–52, 59, 63, 95–97]. However, most of these studies have been case-control (or cross-sectional) in design. In previous prospective analyses in this perinatal cohort, lower maternal vitamin B_12_ concentrations in pregnancy were associated with increased risk of IUGR [57], and lower tertiles of maternal vitamin B_12_ levels throughout pregnancy were associated with increased risk of SGA [67], although vitamin B_12_ deficiency (<148 pmol/L) was not significantly associated with risk of SGA [58]. However, these analyses used the previous definition of SGA and IUGR as <10^th^ percentile of birth weight for gestational age, which constrains comparability of findings. In contrast, other studies have reported that inadequate maternal vitamin B_12_ status during pregnancy was not associated with risk of adverse birth outcomes, such as gestational age at birth [98–100], birth weight [12, 28, 60, 82, 87, 98–111], LBW (<2,500 g) [12, 67], or SGA [60, 98, 105, 106, 109, 111]. In a systematic review and meta-analysis of vitamin B_12_ in pregnancy and preterm birth and low birth weight, maternal vitamin B_12_ levels during pregnancy were not significantly associated with birth weight [58]. In overall meta-analyses, vitamin B_12_ levels were associated with lower risk of preterm birth (ARR 0.89 (95% CI 0.82, 0.97); and low vitamin B_12_ status (<148 pmol/L) was associated with increased risk of low birth weight (<2,500; ARR: 1.15, 95% CI: 1.01, 1.31), although these findings were not significant in the individual studies [58]. Randomized trials are needed to determine the efficacy of vitamin B_12_ supplementation on birth outcomes, including preterm birth, low birth weight, and small-for-gestational age.

In this study, maternal vitamin B_12_ status was not associated with other neonatal outcomes, including WHO z-scores, ponderal index, mid-upper arm circumference, or head circumference. Although few studies have evaluated the association of vitamin B_12_ status during pregnancy and infant anthropometric outcomes, most studies to date have focused on neonatal head circumference, and findings have been heterogenous [61, 88, 99, 102]. Findings regarding the association of maternal vitamin B_12_ status with other infant health outcomes have been divergent.

This study is among the largest prospective studies to date to examine the burden of vitamin B_12_ deficiency in pregnancy and its associations with neonatal vitamin B_12_ status and perinatal outcomes. Maternal vitamin B_12_ biomarkers were evaluated early in pregnancy (~12 weeks’ gestation), and vitamin B_12_ status was evaluated with both circulating (i.e., vitamin B_12_) and functional (i.e., MMA, tHcy) biomarkers. We also considered additional indicators of vitamin B_12_ status, including cB12 and impaired vitamin B_12_ status, which combine circulating and functional biomarkers.

This study has several limitations. Maternal and neonatal vitamin B_12_ status were assessed at a single time point at enrollment and birth, respectively. The assessment of maternal vitamin B_12_ status ~12 weeks of gestation may not reflect vitamin B_12_ status periconceptionally or throughout pregnancy. Assessment of neonatal vitamin B_12_ status using cord blood at birth is an important study limitation–and limits interpretations of the associations between maternal vitamin B_12_ and infant status early in life. Participants in this biomarker sub-study were similar to the overall perinatal cohort (n = 1,272), in terms of sociodemographic status (e.g., age, educational level), gestational age at enrollment, parity, nutritional variables (e.g., weight, BMI, hemoglobin), and birth outcomes (e.g., live birth, preterm delivery, birth weight, LBW, SGA); however, they may differ on other unmeasured variables. In terms of vitamin B_12_ biomarkers, in addition to total vitamin B_12_, MMA, and tHcy, inclusion of holo-transcobalamin may be an important circulating biomarker of vitamin B_12_ status during gestation, although it has not been validated in pregnancy [112]. The role of vitamin B_12_ needs to be examined in the context of folate-mediated one-carbon metabolism. Vitamin B_12_ deficiency may co-occur with other micronutrient deficiencies (e.g., folate) that may influence vitamin B_12_ status and are independent risk factors for adverse perinatal outcomes. An important limitation of this study is the use of vitamin B_12_ biomarker cut-offs from adult non-pregnant populations–these vitamin B_12_ biomarker cut-offs have not been validated in pregnancy or in infancy. Further research is needed to develop and validate vitamin B_12_ biomarker cut-offs in pregnant women and young infants. Although findings provide evidence of an association of maternal and neonatal vitamin B_12_ status, the interpretations of these associations are not causal. Importantly, our study was not powered to detect differences in birth outcomes, including low birth weight and small for gestational age. Randomized trials are needed to examine the effects of vitamin B_12_ (periconceptionally and throughout pregnancy) on the development of maternal and infant health outcomes.

In summary, in this cohort of pregnant women, the prevalence of vitamin B_12_ deficiency was high early in pregnancy and predicted risk of infant vitamin B_12_ deficiency. This is one of the largest studies to date to evaluate the burden of vitamin B_12_ deficiency in pregnant women and their neonates. Findings suggest that vitamin B_12_ deficiency is an important public health problem in this population, and vitamin B_12_ status early in pregnancy has an important role in determining vitamin B_12_ status early in life. Future research, including randomized trials, is needed to determine the independent effects of vitamin B_12_ on the development of perinatal outcomes, to inform screening and interventions to improve maternal and child health.

## Supporting information

S1 Table Associations of vitamin B12 biomarkers with vitamin B12 deficiency in pregnant women and their neonates. (DOCX)

S2 Table Associations between maternal vitamin B12 concentrations at enrollment and perinatal outcomes. (DOCX)

S3 Table Associations between maternal methylmalonic acid concentrations at enrollment and perinatal outcomes. (DOCX)

S4 Table Associations between maternal impaired vitamin B12 status at enrollment and perinatal outcomes. (DOCX)
