# Supplementary material for: Maternal vitamin B12 deficiency and perinatal outcomes in southern India
Source: PLoS One. 2021 Apr 6;16(4):e0248145. doi: 10.1371/journal.pone.0248145 (PMC8023483; doi:10.1371/journal.pone.0248145)
Supplement: S1 Table — (DOCX) [file pone.0248145.s001.docx]

**S1 Table.** Associations of Vitamin B_12_ Biomarkers with Vitamin B_12_ Deficiency in Pregnant Women and their Neonates

| ***Maternal Biomarkers^1,2^*** | **n** | **Vitamin B_12_ <148 pmol/L** | **Vitamin B_12_ ≥148** | **P-value^3^** | **RR (95% CI)** | **P-value** |
| --- | --- | --- | --- | --- | --- | --- |
|  |  |  |  |  |  |  |
| Plasma MMA, µmol/L | 376 | 0.40 (0.26, 0.61) | 0.35 (0.21, 0.58) | 0.02 | 1.12 (1.01, 1.24) | 0.04 |
| >0.26 µmol/L |  | 178 (75.7) | 90 (63.8) | 0.01 | 1.26 (1.03, 1.53) | 0.02 |
| >0.37 µmol/L |  | 130 (55.3) | 64 (45.4) | 0.06 | 1.16 (0.99, 1.36) | 0.06 |
| Plasma tHcy, µmol/L | 377 | 16.48 (12.52, 21.40) | 14.75 (11.89, 19.42) | 0.056 | 1.18 (0.99, 1.42) | 0.07 |
| >15.0 µmol/L |  | 138 (58.7) | 68 (47.9) | 0.04 | 1.18 (1.00, 1.39) | 0.04 |
| >10.0 µmol/L |  | 213 (90.6) | 121 (85.2) | 0.11 | 1.25 (0.92, 1.69) | 0.15 |
|  |  |  |  |  |  |  |
| ***Neonatal Biomarkers^1,2^*** | **n** | **Vitamin B_12_ <148 pmol/L** | **Vitamin B_12_ ≥ 148** | **P-value^3^** | **RR (95% CI)** | **P-value** |
|  |  |  |  |  |  |  |
| Plasma MMA, µmol/L | 231 | 0.71 (0.49, 0.98) | 0.58 (0.44, 0.78) | **0.0001** | 1.59 (1.25, 2.02) | **0.0001** |
| >0.26 µmol/L |  | 126 (96.2) | 191 (95.5) | 0.76 | 1.11 (0.54, 2.28) | 0.77 |
| >0.37 µmol/L |  | 120 (91.6) | 169 (84.5) | 0.06 | 1.59 (0.94, 2.68) | 0.09 |
| Plasma tHcy, µmol/L | 231 | 19.72 (13.96, 26.15) | 16.77 (12.72, 23.63) | 0.01 | 1.37 (1.06, 1.76) | 0.01 |
| >15.0 µmol/L |  | 93 (71.0) | 127 (63.5) | 0.16 | 1.23 (0.91, 1.67) | 0.17 |
| >10.0 µmol/L |  | 123 (93.9) | 174 (87.0) | 0.04 | 1.76 (0.95, 3.27) | 0.07 |

^1^Values are median (IQR) and n (%); ^2^Statistical analyses: Kruskal Wallis and Chi-Sq tests were used for continuous and categorical comparisons, respectively; binomial regression models were used to examine associations between maternal and neonatal biomarker concentrations and vitamin B_12_ deficiency; ^3^Continuous biomarkers were natural logarithmically transformed to achieve normality prior to analysis; *Abbreviations:* MMA, methylmalonic acid; tHcy, total homocysteine.
