# Supplementary material for: Maternal vitamin B12 deficiency and perinatal outcomes in southern India
Source: PLoS One. 2021 Apr 6;16(4):e0248145. doi: 10.1371/journal.pone.0248145 (PMC8023483; doi:10.1371/journal.pone.0248145)
Supplement: S2 Table — (DOCX) [file pone.0248145.s002.docx]

| **S2 Table.** Associations Between Maternal Vitamin B_12_ Concentrations at Enrollment and Perinatal Outcomes | | | | | |
| --- | --- | --- | --- | --- | --- |
|  |  | **Univariate^2^** | | **Multivariate^3^** | |
| **Neonatal Variables^1^** | **n** | **β (SE) or RR (95% CI)** | **P-value** | **β (SE) or RR (95% CI)** | **P-value^6^** |
| Sex, male | 399 | 0.99 (0.85, 1.16) | 0.91 | 0.99 (0.85, 1.16) | 0.94 |
| Birth weight, g | 399 | 7.50 (37.59) | 0.84 | 28.76 (37.36) | 0.44 |
| <2,500 g |  | 0.95 (0.62, 1.44) | 0.37 | 0.91 (0.62, 1.35) | 0.65 |
| Gestational age at birth, wks | 399 | –0.09 (0.12) | 0.45 | –0.05 (0.12) | 0.71 |
| <37 wks |  | 1.06 (0.54, 2.09) | 0.73 | 1.03 (0.52, 2.02) | 0.94 |
| Small for gestational age^4^ | 399 | 0.88 (0.64, 1.21) | 0.20 | 0.82 (0.60, 1.12) | 0.21 |
| Hemoglobin, g/dL | 252 | –0.14 (0.31) | 0.65 | –0.02 (0.32) | 0.95 |
| <11.0 g/dL |  | 1.44 (0.92, 2.26) | 0.11 | 1.51 (0.90, 2.52) | 0.12 |
| Birth length, cm | 389 | –0.06 (0.17) | 0.70 | 0.03 (0.17) | 0.87 |
| Ponderal index^5^, g/cm^3^ | 389 | 0.0002 (0.0002) | 0.38 | 0.0003 (0.0002) | 0.30 |
| Length-for-age z-score (LAZ) | 389 | –0.03 (0.09) | 0.77 | 0.02 (0.09) | 0.80 |
| Stunting (LAZ <–2) |  | 0.71 (0.30, 1.72) | 0.45 | 0.67 (0.31, 1.47) | 0.32 |
| Weight-for-age z-score (WAZ) | 399 | 0.02 (0.09) | 0.79 | 0.07 (0.09) | 0.41 |
| Underweight (WAZ <–2) |  | 0.93 (0.56, 1.56) | 0.79 | 0.88 (0.54, 1.44) | 0.61 |
| Weight-for-length z-score (WLZ) | 384 | 0.10 (0.12) | 0.37 | 0.12 (0.12) | 0.31 |
| Wasting (WLZ <–2) |  | 0.93 (0.74, 1.18) | 0.56 | 0.93 (0.74, 1.17) | 0.55 |
| Head circumference, cm | 391 | 0.05 (0.11) | 0.67 | 0.11 (0.11) | 0.33 |
| Chest circumference, cm | 390 | –0.02 (0.16) | 0.88 | 0.05 (0.16) | 0.74 |
| Mid-upper arm circumference, cm | 391 | –0.003 (0.08) | 0.97 | 0.03 (0.08) | 0.68 |
| Biceps skinfold, mm | 390 | 0.03 (0.06) | 0.62 | 0.04 (0.06) | 0.47 |
| Triceps skinfold, mm | 390 | –0.01 (0.07) | 0.88 | 0.03 (0.07) | 0.73 |
| Subscapular skinfold, mm | 390 | –0.06 (0.08) | 0.47 | –0.03 (0.08) | 0.75 |

^1^ Statistical analyses: linear regression or binomial regression models were used to examine associations between maternal vitamin B_12_ concentrations and perinatal outcomes. Poisson regression models were used when binomial models did not converge. Maternal biomarkers were natural logarithmically transformed to achieve normality prior to analysis; ^2^Adjusted for gestational age at enrollment; ^3^Adjusted for gestational age at enrollment, parity, and maternal age in years, BMI, and educational level; ^4^ Small for gestational age (SGA) was defined as birth weight <10^th^ percentile for gestational age and sex, using INTERGROWTH [73]; ^5^ Neonatal ponderal index was calculated as the ratio of weight to length (g/cm^3^ × 100). ^6*^After adjusting for multiple hypothesis testing, associations were considered significant if p<0.002.
