# Supplementary material for: Maternal vitamin B12 deficiency and perinatal outcomes in southern India
Source: PLoS One. 2021 Apr 6;16(4):e0248145. doi: 10.1371/journal.pone.0248145 (PMC8023483; doi:10.1371/journal.pone.0248145)
Supplement: S3 Table — (DOCX) [file pone.0248145.s003.docx]

**S3 Table.** Associations Between Maternal Methylmalonic Acid Concentrations at Enrollment and Perinatal Outcomes

|  |  | **Univariate^2^** | | **Multivariate^3^** | |
| --- | --- | --- | --- | --- | --- |
| **Neonatal Variables**^1^ | **n** | **β (SE) or RR (95% CI)** | **P-value** | **β (SE) or RR (95% CI)** | **P-value^6^** |
| Sex, male | 376 | 0.91 (0.78, 1.06) | 0.21 | 0.90 (0.78, 1.05) | 0.17 |
| Birth weight, g | 376 | –18.37 (33.51) | 0.58 | –19.77 (32.90) | 0.55 |
| <2,500 g |  | 0.98 (0.67, 1.43) | 0.93 | 0.97 (0.67, 1.42) | 0.89 |
| Gestational age at birth, wks | 376 | 0.04 (0.11) | 0.74 | 0.05 (0.11) | 0.62 |
| <37 wks |  | 1.24 (0.67, 2.31) | 0.49 | 1.23 (0.71, 2.13) | 0.47 |
| Small for gestational age^4^ | 376 | 1.04 (0.77, 1.39) | 0.81 | 1.03 (0.77, 1.39) | 0.83 |
| Hemoglobin, g/dL | 239 | –0.01 (0.29) | 0.96 | 0.05 (0.28) | 0.86 |
| <11.0 g/dL |  | 0.98 (0.62, 1.54) | 0.92 | 0.96 (0.60, 1.54) | 0.88 |
| Birth length, cm | 368 | –0.03 (0.15) | 0.84 | –0.02 (0.14) | 0.88 |
| Ponderal index^5^, g/cm^3^ | 368 | –0.0001 (0.0002) | 0.68 | –0.0001 (0.0002) | 0.62 |
| Length-for-age z-score (LAZ) | 368 | 0.004 (0.08) | 0.96 | 0.01 (0.08) | 0.90 |
| Stunting (LAZ <–2) |  | 1.52 (0.69, 3.34) | 0.30 | 1.47 (0.67, 3.22) | 0.34 |
| Weight-for-age z-score (WAZ) | 376 | –0.03 (0.08) | 0.70 | –0.03 (0.08) | 0.68 |
| Underweight (WAZ <–2) |  | 1.01 (0.64, 1.60) | 0.96 | 1.02 (0.67, 1.55) | 0.93 |
| Weight-for-length z-score (WLZ) | 364 | –0.04 (0.10) | 0.74 | –0.04 (0.10) | 0.67 |
| Wasting (WLZ <–2) |  | 0.98 (0.80, 1.21) | 0.87 | 1.02 (0.82, 1.26) | 0.88 |
| Head circumference, cm | 370 | –0.005 (0.10) | 0.96 | –0.002 (0.09) | 0.98 |
| Chest circumference, cm | 369 | 0.08 (0.14) | 0.55 | 0.09 (0.14) | 0.52 |
| Mid-upper arm circumference, cm | 370 | –0.01 (0.07) | 0.91 | –0.01 (0.07) | 0.89 |
| Biceps skinfold, mm | 369 | 0.02 (0.05) | 0.73 | 0.01 (0.05) | 0.77 |
| Triceps skinfold, mm | 369 | 0.01 (0.06) | 0.93 | 0.01 (0.06) | 0.92 |
| Subscapular skinfold, mm | 369 | 0.01 (0.07) | 0.89 | 0.01 (0.07) | 0.92 |

^1^Statistical analyses: linear regression or binomial regression models were used to examine associations between maternal biomarkers and perinatal outcomes. Poisson regression models were used when binomial models did not converge. Maternal methylmalonic acid was natural logarithmically transformed to achieve normality prior to analysis; ^2^Adjusted for gestational age at enrollment; ^3^Adjusted for gestational age at enrollment, parity, and maternal age in years, BMI, and educational level; ^4^ Small for gestational age (SGA) was defined as birth weight <10^th^ percentile for gestational age and sex, using INTERGROWTH [73]; ^5^ Neonatal ponderal index was calculated as the ratio of weight to length (g/cm^3^ × 100). ^6*^After adjusting for multiple hypothesis testing, associations were considered significant if p<0.002.
