# Supplementary material for: Maternal vitamin B12 deficiency and perinatal outcomes in southern India
Source: PLoS One. 2021 Apr 6;16(4):e0248145. doi: 10.1371/journal.pone.0248145 (PMC8023483; doi:10.1371/journal.pone.0248145)
Supplement: S4 Table — (DOCX) [file pone.0248145.s004.docx]

**S4 Table.** Associations Between Maternal Impaired Vitamin B_12_ Status at Enrollment and Perinatal Outcomes

|  |  | **Univariate^2^** | | **Multivariate^3^** | |
| --- | --- | --- | --- | --- | --- |
| **Neonatal Variables^1^** | **n** | **β (SE) or RR (95% CI)** | **P-value** | **β (SE) or RR (95% CI)** | **P-value^6^** |
| Sex, male | 376 | 0.95 (0.78, 1.16) | 0.64 | 0.95 (0.78, 1.16) | 0.62 |
| Birth weight, g | 376 | –4.05 (44.31) | 0.93 | –12.50 (43.64) | 0.77 |
| <2,500 g |  | 1.07 (0.64, 1.78) | 0.79 | 1.06 (0.64, 1.75) | 0.83 |
| Gestational age at birth, wks | 376 | 0.23 (0.14) | 0.11 | 0.21 (0.14) | 0.15 |
| <37 wks |  | 0.77 (0.34, 1.75) | 0.52 | 0.78 (0.34, 1.77) | 0.55 |
| Small for gestational age^4^ | 376 | 0.94 (0.64, 1.38) | 0.74 | 0.96 (0.65, 1.42) | 0.85 |
| Hemoglobin, g/dL | 239 | 0.40 (0.38) | 0.30 | 0.38 (0.38) | 0.32 |
| <11.0 g/dL |  | 0.49 (0.25, 0.94) | **0.03** | 0.47 (0.24, 0.91) | 0.02 |
| Birth length, cm | 368 | –0.13 (0.19) | 0.48 | –0.16 (0.19) | 0.41 |
| Ponderal index^5^, g/cm^3^ | 368 | 0.0001 (0.0003) | 0.63 | 0.00 (0.00) | 0.68 |
| Length-for-age z-score (LAZ) | 368 | –0.07 (0.10) | 0.50 | –0.08 (0.10) | 0.43 |
| Stunting (LAZ <–2) |  | 2.00 (0.68, 5.85) | 0.21 | 2.12 (0.73, 6.15) | 0.17 |
| Weight-for-age z-score (WAZ) | 376 | –0.01 (0.10) | 0.96 | –0.02 (0.10) | 0.81 |
| Underweight (WAZ <–2) |  | 1.11 (0.60, 2.07) | 0.74 | 1.12 (0.60, 2.09) | 0.72 |
| Weight-for-length z-score (WLZ) | 364 | 0.05 (0.14) | 0.70 | 0.04 (0.14) | 0.76 |
| Wasting (WLZ <–2) |  | 0.86 (0.64, 1.14) | 0.29 | 0.87 (0.65, 1.16) | 0.33 |
| Head circumference, cm | 370 | –0.06 (0.13) | 0.61 | –0.10 (0.13) | 0.41 |
| Chest circumference, cm | 369 | –0.07 (0.18) | 0.69 | –0.12 (0.18) | 0.51 |
| Mid-upper arm circumference, cm | 370 | –0.05 (0.09) | 0.59 | –0.07 (0.09) | 0.42 |
| Biceps skinfold, mm | 369 | 0.01 (0.07) | 0.86 | 0.00 (0.07) | 0.94 |
| Triceps skinfold, mm | 369 | 0.03 (0.08) | 0.71 | 0.02 (0.08) | 0.84 |
| Subscapular skinfold, mm | 369 | 0.07 (0.09) | 0.49 | 0.05 (0.09) | 0.59 |

^1^Statistical analyses: linear regression or binomial regression models were used to examine associations between maternal biomarkers and perinatal. Poisson regression models were used when binomial regression models did not converge; ^2^ Adjusted for gestational age at enrollment; ^3^ Adjusted for gestational age at enrollment, parity, and maternal age in years, BMI, and educational level; ^4^ Small for gestational age (SGA) was defined as birth weight <10^th^ percentile for gestational age and sex, using INTERGROWTH [73]; ^5^Neonatal ponderal index was calculated as the ratio of weight to length (g/cm^3^ × 100). ^6*^After adjusting for multiple hypothesis testing, associations were considered significant if p<0.002.
